# High-Intensity Interval Circuit Training Versus Moderate-Intensity Continuous Training on Cardiorespiratory Fitness in Middle-Aged and Older Women: A Randomized Controlled Trial

**DOI:** 10.3390/ijerph17051805

**Published:** 2020-03-10

**Authors:** Ismael Ballesta-García, Ignacio Martínez-González-Moro, Domingo J Ramos-Campo, María Carrasco-Poyatos

**Affiliations:** 1 Physical Exercise and Human Performance Research Group, University of Murcia, 30003 Murcia, Spain; Ismael.b.g@um.es (I.B.-G.); igmartgm@um.es (I.M.-G.-M.); 2 Department of Physiotherapy, University of Murcia, 30003 Murcia, Spain; 3 Department of Physical Activity and Sport Sciences, UCAM Research Centre for High Performance Sport, Catholic University of San Antonio de Murcia, 30107 Murcia, Spain; djramos@ucam.edu; 4 Department of Education, Health and Public Administration Research Center, University of Almeria, 04120 Almeria, Spain

**Keywords:** high-intensity interval circuit training, peak oxygen consumption, middle-aged, older, women

## Abstract

High-intensity interval training (HIIT) has similar or better effects than moderate-intensity continuous training (MICT) in increasing peak oxygen consumption (VO_2max_), however, it has not been studied when HIIT is applied in a circuit (HIICT). The aim of this study was to compare the effects of a HIICT versus MICT on VO_2max_ estimated (VO_2max_-ES), heart rate (HR) and blood pressure (BP) of middle-aged and older women. A quasi-experimental randomized controlled trial was used. Fifty-four women (67.8 ± 6.2 years) were randomized to either HIICT (*n* = 18), MICT (*n* = 18) or non-exercise control group (CG; *n* = 18) for 18 weeks. Participants in HIICT and MICT trained two days/week (one hour/session). Forty-one participants were assessed (HIICT; *n* = 17, MICT; *n* = 12, CG; *n* = 12). Five adverse events were reported. Cardiorespiratory fitness, HR and BP were measured. The tests were performed before and after the exercise intervention programs. VO_2max_-ES showed significant training x group interaction, in which HIICT and MICT were statistically superior to CG. Moreover, HIICT and MICT were statistically better than CG in the diastolic blood pressure after exercise (DBP_ex_) interaction. For the systolic blood pressure after exercise (SBP_ex_), HIICT was statistically better than CG. In conclusion, both HIICT and MICT generated adaptations in VO_2max_-ES and DBP_ex_. Furthermore, only HIICT generated positive effects on the SBP_ex_. Therefore, both training methods can be considered for use in exercise programs involving middle-aged and older women.

## 1. Introduction

The over-65-year-old population has increased rapidly in recent years [1]. Aging is accompanied by the development of chronic diseases, which makes it necessary to give greater attention to the elderly population [2]. Maximal oxygen consumption (VO_2max_) is recognized as one of the most important indicators in the prognosis of good health in older people [3] and it is used to predict survival in patients with cardiovascular disease [4,5,6,7,8]. A decrease in VO_2max_ is one of the clearest consequences of aging since, from the age of 30, it decreases by 10% every 10 years in people with a sedentary lifestyle [9]. In addition, from the age of 60, this decrease in VO_2max_ is associated with reduced functional ability, a component related to the autonomy to carry out activities in daily life (i.e., walking or climbing stairs) [2]. Because of hormonal changes (i.e., menopause), it is more difficult to control this decrease in VO_2max_ in middle-aged and older women [9].

Nowadays, it is known that aerobic exercise is essential for maintaining health and functional capacity in this population [10,11,12]. In fact, many studies indicate that maintaining good cardiorespiratory fitness should be one of the main goals in women’s exercise programs [13,14,15]. Consequently, governments and healthcare systems are significantly increasing the number of physical exercise programs directed at this population. One of the principal characteristics of these programs is that the training is done on a circuit. Circuit training consists of exercises involving all the muscle groups and it aims to simultaneously improve strength and cardiorespiratory fitness [16,17].

Research has indicated that moderate-intensity circuit training (MICT) improves VO_2_ peak [18], blood pressure (BP) [19], and heart rate (HR) [20] in middle-aged and older people. However, high-intensity interval training (HIIT) has emerged as an alternative training method, remarkably in cardiac rehabilitation programs [21]. HIIT includes intervals of high-intensity aerobic exercise (85%–100% of VO_2max_) interspersed with periods of relative rest (0%–40% of VO_2max_) [22]. In fact, much research has shown strong evidence that HIIT is an effective method for improving VO_2_ peak [21,23], BP [24,25] and post-exercise HR recovery [18,26] in people with cardiovascular diseases. Indeed, the literature shows a more favorable trend for HIIT than for MICT in improving health indices and markers [22].

Nonetheless, although the literature supports the benefits of HIIT in healthy middle-aged and older women, HIIT methodology (HIICT) has never been applied to circuit training in this population. HIICT may be an alternative training method for improving cardiorespiratory fitness and performance in daily activities due to integrated global multiplanar-based movements (squatting, pulling, etc.) performed at high speed, which makes it functional and highly transferable in older adults [27,28].

Therefore, our main objective was to compare the effects of HIICT versus MICT on the maximal oxygen consumption (VO_2max_), heart rate (HR), and blood pressure (BP) of middle-aged and older women. Based on previous research, our hypothesis was that both HIICT and MICT would significantly improve the VO_2max_, HR parameters, and BP in this population, with greater adaptations in the HIICT group.

## 2. Materials and Methods

### 2.1. Design 

This 18-weeks quasi-experimental randomized controlled trial was registered prospectively with ClinicalTrials.gov (NCT03840330). The participants who qualified to participate in the study were randomly allocated to either a HIICT group (*n* = 18), a MICT group (*n* = 18), or a no-exercise control group (CG; *n* = 18). The trial design followed CONSORT guidelines and was approved by the University of Almería Bioethics Committee (UALBIO2019/006).

### 2.2. Participants

Ninety middle-aged and older women (67.8 ± 6.2 years) were invited to participate in the study. Recruitment took place between September 2017 and December 2017 from elderly day-care centers in Murcia (Spain). Prior to the beginning of the study, invited participants signed a consent form and performed a general medical evaluation to ensure that they were physically and mentally capable of following the training programs. As inclusion criteria, participants had to be women aged between 55 and 85 years, have full autonomy in their daily activities in accordance with the Lawton and Brody [29] and Katz [30] scales, have given no positive responses in the Physical Activity Readiness Questionnaire or a positive response to Item 6. This, therefore, included women with controlled hypertension but not women with cardiac, respiratory or joint diseases. Women who were participating or had previously participated in a training program with similar exercises over the last three months, and women with uncontrolled hypertension, were also excluded. Participants had to complete at least 80% of the training sessions.

### 2.3. Interventions 

Women in the intervention groups (HIICT and MICT) were required to train twice a week for 18 weeks, with each session lasting one hour. The intervention was implemented from January to May 2018. Participants assigned to the CG were encouraged to maintain their normal physical-activity habits. A graduate in Physical Activity and Sport Sciences directed all training sessions.

After a two-week familiarization period, the training programs were divided into four four-week mesocycles designed to be progressively more challenging. Each session was divided into (1) a warm-up period, (2) the main HIICT or MICT exercise programs, and (3) a cool-down period. The 6- to 20-points Borg scale of perceived exertion was used to control the training intensity [11]. The HIICT group exercises were performed at high speeds with the objective of reaching a 12–18-point rating of perceived exertion (RPE), while the MICT group exercises were performed at moderate speeds, the objective being to reach a 6–14 points RPE rating. An example of the training sessions and intensity progression that was implemented is given by Ballesta-García et al. [31].

### 2.4. Outcomes

The estimation of VO_2max_ (VO_2max_-ES) was the primary outcome of this study, while the BP, HR, and maximal speed reached during the treadmill test were the secondary outcomes. The tests were performed on all participants before and after the exercise intervention programs. The pre-tests were done in January (2018) and the post-test was carried out in May (2018) over a one-week period.

The cardiorespiratory fitness test was performed on a treadmill (RUN 7411 Elite-PC, Runner, Cavezzo, Italy). A modified Bruce protocol graded exercise was used [32]. This is a triangular test in which the load was gradually increased every three minutes. The participants were encouraged to perform maximal exercise. Their maximal effort was verified when the maximum HR exceeded 85% of their theoretical maximum (220 - age). The test continued until the participants were exhausted, which was generally due to muscle and/or respiratory fatigue. Recovery was performed at 40% of the maximum peak exercise velocity and at a gradient of 0º. The VO_2max_-ES was calculated from the formula described in the ACSM guidelines [33]. The maximal speeds reached were recorded.

HR parameters were monitored with a 12-lead electrocardiograph (Cardioline RealClick PC-based ECG, Trento, Italy). They were taken: 1) Every minute during the treadmill test and 2) a minute after finishing the treadmill test (HR_rec_).

BP was checked with a sphygmomanometer (NCD Medical, Prestige medical 80, Dublin, Ireland) and a stethoscope at two different points in time: (1) Just after the end of treadmill test (BP_ex_) and (2) one minute after the treadmill test (BP_rec_).

Height and weight were measured using an electronic balance and a height rod (Seca 768), respectively, and body mass index (BMI) was calculated according to the formula: BMI = kg/m^2^.

### 2.5. Sample size and Power

Rstudio 3.15.0 software was used for calculating sample size. The significance level was accepted at *p* ≤ 0.05. According to the mean standard deviation established for the VO_2max_ test in previous studies [34,35] (SD = 5.4 ml/kg/min) and an estimated error (*d*) of 1.45 mL/kg/min, a valid sample size for a 95% confidence interval (CI) was 53 (*n* = *CI*^2^ × *d*^2^/*SD*^2^). Forty-one women completed the study. The final sample size obtained for each group in our study (HIICT = 17, MICT = 12, CG = 12) gave powers of 81%, 65%, and 65%, respectively, if between and within a variance of 1.

### 2.6. Randomization and Blinding

All participants were assigned randomly into the groups in equal sample sizes (HIICT, MICT and CG, *n* = 18), with a block randomization method. Block size was determined based on the statistical power provided. Following Kim and Shin [36], Excel 2016 (Microsoft, Redmond, WA, USA) was used to create a randomization sequence. Randomization was performed in a 1:1 allocation via a random number table. Owing to the difficulty of blinding participants and instructors in the exercise trials, only the research staff performing the assessment and statistical analysis were blinded to the group assignment. Central allocation was the allocation concealment method selected.

### 2.7. Statistical Analysis

Jamovi 1.2.5 software (Jamovi Project 2018) and Rstudio 3.15.0 software (Rstudio inc., Boston, MA, USA) were used for data analysis. The normality of distribution was tested using the Kolmogorov–Smirnov test. Levene’s test was performed to determine the homogeneity of variance. The statistical analysis was performed according to the intention-to-treat (ITT) principle (last observation carried forward). Descriptive data are reported as mean ± SD and range. To compare variables before the intervention, analysis of variance (ANOVA) for repeated measures was calculated (the general linear model). The analysis of covariance (ANCOVA) was used to compare variables after the intervention. Baseline values were included as co-variables in order to adjust for potential baseline differences in the dependent variables. The age was also included as a co-variable because of the wide range considered in the present study (55–85 years). Cohen´s effect size (ES) statistic and 95% confidence intervals (CI) were calculated to determine an ES difference [37]. Statistical significance was accepted at *p* ≤ 0.05.

## 3. Results

**Figure 1:**
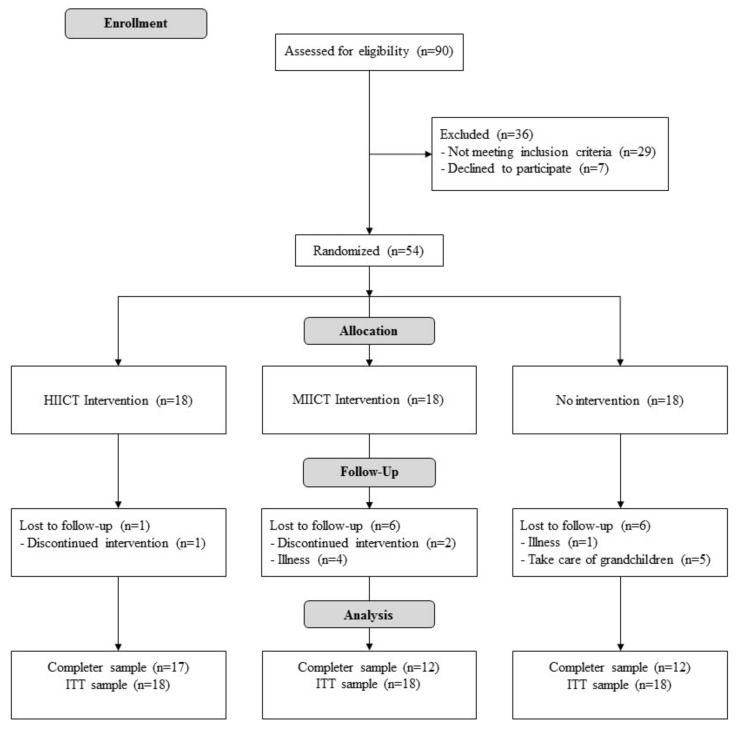
Study flow chart.

The flow diagram is shown in Figure 1. Thirty-six women were not included in the study. A total of fifty-four participants were enrolled in the study and randomly distributed into HIICT, MICT, and CG. Finally, forty-one women (HIICT, *n* = 17; MICT, *n* = 12; GC, *n* = 12) completed the study. The study was completed in May 2018. Table 1 summarizes the baseline characteristics of the participants.

### 3.1. Inter-group Results

The inter-group results for the primary and secondary outcomes are presented in Table 2. The principal analysis of these results indicated that there was a significant training x group interaction in the VO_2max_-ES (*p* = 0.002, F = 7.36, ES = 0.224), the SBP_ex_ (*p* = 0.038, F = 3.48, ES = 0.120), the DBP_ex_ (*p* = <0.001, F = 17.4, ES = 0.405) and the maximal speed reached during treadmill test (*p* = 0.001).

- For VO_2max_-ES, HIICT was statistically superior to the CG (dif = 3.4 ml/kg/min, *t* = −3.73) and MICT was also statistically superior to the CG (dif = 1.9 ml/kg/min, *t* = −2.65).
- For SBP_ex_, HIICT was statistically better than the CG (dif = −6,39 mmHg, t = −0.122).
- For DBP_ex_, HIICT was statistically better than the CG (dif = −5,00 mmHg, *t* = −3.933) and MICT was also statistically better than the CG (dif = −7,50 mmHg, *t* = 3.989).
- Finally, for maximal speed reached during treadmill test, HIICT was statistically superior to the CG (dif = 0.2 m/s, *t* = −1.96) and MICT was also statistically superior to the CG (dif = 0.57 m/s, *t* = −2.96).

### 3.2. Intra-group Results

The intra-group analysis (Table 3) showed a significant improvement in the VO_2max_-ES, and the maximal speed reached during the treadmill test for both HIICT (*p* < 0.001) and MICT (*p* < 0.010 and *p* < 0.015, respectively). Regarding the SBP_ex_, either MICT or the CG indicated a significant decrease (*p* = 0.015 and *p* = 0.015, respectively). Instead, a significant increase was observed in the SBP_ex_ for HIICT and MICT (*p* < 0.003 and *p* = 0.002, respectively), as well as a significant decrease for the CG (*p* = 0.002).

Regarding safety, five women (four in MICT and one in the CG) presented adverse events. None of these adverse events occurred during the training sessions (one eye surgery, foot surgery, clavicle fracture, and two hip fractures after a fall).

## 4. Discussion

The aim of this trial was to investigate what type of training (HIICT or MICT) produces improved adaptations in VO_2max_-ES, HR parameters and BP of middle-aged and older women. The findings regarding the primary outcome suggest that both high- and moderate-intensity circuit training led to significant adaptations in the VO_2max_-ES. In addition, both the HR_ex_ and the one-minute HR recovery after the treadmill test remained unchanged in all groups. Moreover, SBP_ex_ and DBP_ex_ were better in HIICT than in the control group. Finally, the maximal speed reached in the treadmill test was higher in HIICT and MICT than in the control group. These results highlight that HIICT and MICT could play a leading role in the maintenance of good health in middle-aged and older women, although the differentiating effect of HIICT relies on the need to apply a lower total workload, as evidenced by Ballesta et al. [31].

According to VO_2max_-ES results, our study showed a significant improvement in the treadmill test after the 18-week training period for both the HIICT and MICT groups, with significant differences to the CG. Although there were no significant differences between HIICT and MICT, a higher effect size was obtained in HIICT (ES = 0.58) compared to MICT (ES = 0.32). Therefore, there seems to be a greater trend for VO_2max_-ES improvement in HIIT than in MICT. Our results are in accordance with various studies showing that the application of both HIICT and MICT succeeded in increasing the VO_2max_ values for subjects with cardiovascular diseases [4,24], as well as in healthy elderly and middle-aged people [38]. Similarly, in line with the better trend shown by HIICT, a meta-analysis showed that HIIT improved VO_2max_ more than MICT in overweight or obese adults [39]. Despite the fact that our study measured the VO_2max_ estimated, the tendency of the results was in the same line. It is not clear why there was no significant difference in VO_2max_-ES between HIICT and MICT in our study. The absence of differences might be due to the fact that the HIICT group did not manage to reach a higher lower limbs speed during training due to the duality of movement and complexity of tasks. This manifested in a similar strength gain to that reported by Ballesta-García et al. [31]. On the other hand, in a recently published meta-analysis, Muñoz-Martínez et al. [16] also cite the effectiveness of circuit training on VO_2max_. On this matter, Chicharro et al. [22] and Muñoz-Martínez et al. [16] indicated that both HIIT and circuit training increase VO_2max_ as a consequence of central and peripheral adaptations, mainly due to increased cardiac output. Therefore, the training methodology carried out in our study could be the cause of the results obtained for VO_2max_-ES.

Consequently, given that there were no significant changes in the HR peak, we could say that the increase in VO_2max_-ES could be related to changes in the stroke volume. These results are in line with those shown by different studies. For example, Wisløff et al. [8] obtained a significant increase in cardiac output, stroke volume, and VO_2_ peak after a 12-week period of HIIT, with no changes in the HR peak. However, Connolly et al. [40] showed that HIIT, not MICT, was effective at increasing the HR peak achieved during a treadmill test in healthy premenopausal women. An animal study concludes that this response appears to be linked to cellular adaptations, such as the rate of Ca^2+^ cycle and the Ca^2+^ sensitivity of cardiomyocytes, produced by HIIT [41]. The relevance of our results on HR peak parameters comes from the fact that different studies have identified its reduction as being the mechanism that causes a decrease in VO_2max_ with aging [9]. Indeed, our results showed that age had an influence on the VO_2max_-ES, with less variation in the group that had older participants (MICT group). With regard to the HR recovery rate, our results are in line with those reflected in the literature, since no intervention group obtained significant changes. In contrast to our results, Villelabeitia-Jaureguizar et al. [18] achieved faster recoveries in coronary heart-disease patients undergoing HIIT at one and two minutes after a treadmill test. However, this did not happen in the MICT group. This result could be associated with both improved endothelial function and decreased pro-inflammatory response during exercise. One study suggests that, unlike in young people, physical exercise is not able to maintain correct autonomic nervous system activation in middle-aged and older women [42]. In this regard, our results went further because there is a relationship between HR recovery and mortality risk [43,44]. Akyüz et al. [45] state that when ≤ 21 bpm is recovered after the first minute of the treadmill test, it is an indicator of the coronary artery disease (CAD) risk. Considering our results in terms of HR peak and HR recovery, we could say that physical exercise could contribute to better cardiovascular health. However, HR results must be treated with caution. Their interpretation is complex because the variable can be influenced by both internal and external factors [20].

Regarding the analysis results for BP_ex_, HIICT was significantly different from CG both SBP_ex_ and DBP_ex_, while the MICT group only obtained significant differences with respect to CG at DBPex. In accordance with our results, Tanaka et al. [46] suggest that endurance-trained subjects manage to achieve higher maximal SBP values. Similarly, Villelabeitia-Jaureguizar et al. [18] showed significant differences between HIIT and MICT after an eight-week training program in CAD patients. Although the mechanisms for increasing SBP during exercise are not clear, a possible explanation for the results obtained might be the workload achieved by the HIIT group during the treadmill test. Tanaka et al. [46] suggest that achieving higher SBP values is a normal adaptive response to increased cardiac output in subjects trained in aerobic endurance, which would be consistent with our VO_2max_-ES results. Regarding the MICT results, and according to the significant interaction of age obtained in this variable, the fact that this was the oldest group may be the reason for the significant decrease in their SBP_ex_ values [47,48]. With regard to the DBP_ex_, Chicharro et al. [49] argue that a decrease in peripheral vascular resistance caused by vasodilation can reduce maximal DBP within certain physiological limits even though this is an anomalous response. Given that all our groups presented maximal DBP hypotension during the treadmill test, the significant increase obtained by the HIICT and MICT groups is a positive effect because the participants maintained a more stable DBP_ex_. Likewise, the significant decrease obtained by the CG could be a consequence of their effort. These results are highly relevant since abnormal DBP response during exercise is associated with an increase in cardiovascular events and mortality [50].

Even though there were no significant differences in SBP or DBP post-exercise recovery between the groups, there was a significant decrease in SBP post-exercise recovery for all of them. However, hypotension below the initial test values did not occur in any of the groups. As indicated by Chicharro et al. [49], there is a rapid drop in both SBP and DBP after exercise. Le et al. [51], on the other hand, indicate that when SBP falls below rest levels, it is a predictive indicator of cardiovascular events. Therefore, although there is a significant reduction in SBP following exercise, it is not an abnormal response. In short, the BP results obtained indicate the positive effect on the cardiovascular health of aerobic physical exercise (especially high-intensity exercise) in middle-aged and older women.

Finally, the maximal speed reached on the treadmill test increased significantly in both HIICT and MICT groups (*p* = 0.001 and *p* = 0.015, respectively). These results are in line with the increase in VO_2max_-ES. Furthermore, these results could be related to an improved acid-base balance at maximal intensities, as pointed out by Villelabeitia-Jauregizar et al. [18]. On the other hand, in the same line as Ayabe et al. [52], our results showed no interaction between maximal speed reached on the treadmill test and BMI.

The force of this research was demonstrating the positive effects of HIICT and MICT on VO_2max_-ES and DBP_ex_ in healthy middle-aged and older women. The clinical implications of the present study relate to the importance of HIICT and MICT as potentially effective methods for improving cardiovascular health in middle-aged and older women, and consequently, on their autonomy to carry out daily activities and on their improved quality of life. In addition, the feasibility of this kind of circuit training means that it can be easily implemented and requires only low-cost materials.

There are several limitations of this study that are worth mentioning—firstly, the use of the estimation of VO_2max_ by ACSM’s equation, since we did not have a gas analyzer to measure it directly, secondly, the wide age range of the sample and the non-blinding of participants and instructors, thirdly, this trial included only a small number of participants, whereas a larger sample size would have helped to quantify the changes resulting from this exercise training more accurately, fourthly, the difficulty that our methodology faced in controlling that all the participants were successfully encouraged to achieve the planned intensity, and finally, the use of the Borg scale to assess exercise intensity. Although the HR is more accurate, it was only used during the familiarization phase because the Borg scale is a more useful and practical tool to guide exercise intensity in daily practice.

## 5. Conclusions

The results of this study suggest that both HIICT and MICT are an effective training method for improving VO_2max_-ES, DBP_ex,_ and maximal speed reached on the treadmill test. On the other hand, HIICT generated better adaptations for the SBP_ex_ than did CG. These results contribute to improved autonomy in carrying out daily activities as well as in preventing the risk of cardiovascular diseases in middle-aged and older women. Our results also reflect the importance of HIICT and MICT in maintaining the health and quality of life of this population.

## Figures and Tables

**Table 1:** Characteristics at baseline (n = 54).

Group	n	Mean	SD	Min	Max	*p*
**Age (years)**						
CG	18	67.4	5.71	59	75	0.370
MICT	18	70	8.76	55	86	
HIICT	18	66.3	5.44	57	76	
**Body Mass Index (kg/m2)**						
CG	18	31.2	4.89	20.9	38.4	0.689
MICT	18	30.1	3.08	24.3	35.9	
HIICT	18	30.4	4.13	35.2	37.7	
**Maximal Oxygen Consumption estimated (ml/kg/min)**						
CG	18	26.8	5.17	15.5	39.0	0.065
MICT	18	25.0	5.57	15.5	33.1	
HIICT	18	26.1	5.63	18.4	36.1	
**Heart Rate peak (bpm)**						
CG	18	144.94	15.4	108	170	0.719
MICT	18	150.78	22.6	119	193	
HIICT	18	148.83	17.7	93	173	
**Heart Rate recovery (bpm)**						
CG	18	79.5	8.1	68.2	91.3	<0.001
MICT	18	85	14.3	60.5	121	
HIICT	18	100	53.0	54.8	296	
**Systolic Blood Pressure exercise (mmHg)**						
CG	18	184	23.50	146	210	0.008
MICT	18	185	10.80	170	210	
HIICT	18	179	9.67	160	200	
**Diastolic Blood Pressure exercise (mmHg)**						
CG	18	76.9	13.2	50	95	0.003
MICT	18	56.9	9.3	40	75	
HIICT	18	58.3	6.9	50	75	
**Systolic Blood Pressure recovery (mmHg)**						
CG	18	152	16.2	120	175	0.042
MICT	18	156	13.6	140	190	
HIICT	18	151	11.7	135	180	
**Diastolic Blood Pressure recovery (mmHg)**						
CG	18	73.3	7.28	65	90	0.001
MICT	18	73.9	7.19	60	85	
HIICT	18	73.9	5.02	65	80	
**Maximal speed reached during treadmill test (m/s)**						
CG	18	4.94	0.809	3.2	6.7	0.054
MICT	18	4.66	0.899	3.2	5.9	
HIICT	18	4.86	0.910	3.7	6.4	

CG—Control Group, MICT—Moderate-Intensity Circuit Training, HIICT—High-Intensity Interval Circuit Training.

**Table 2:** ANCOVA interactions on HIICT, MICT, and CG.

Group			Increment		ANCOVA interactions (F, *p*, ES η²)								
	n (ITT)	n (treated)	Mean	SD	Training x Group			Training x Baseline			Training x Age		
					F	*p*	ES η²	F	*p*	ES η²	F	*p*	ES η²
**Maximal Oxygen Consumption estimated (ml/kg/min)**													
CG	18	12	−0.80	−0.31	7.36	0.002 ^1,2^	0.224	13.60	<0.001	0.171	0.11	0.742	0.002
MICT	18	12	1.90	−0.85									
HIICT	18	17	3.40	0.12									
**Heart Rate peak (bpm)**													
CG	18	12	−4.33	−0.06	3.26	0.474	0.002	2.56	0.115	0.043	8.18	0.006	0.070
MICT	18	12	−0.72	−4.70									
HIICT	18	17	−4.72	2.61									
**Heart Rate recovery (bpm)**													
CG	18	12	0.81	0.00	1.92	0.156	0.070	37.844	<0.001	0.426	0.039	0.844	0.001
MICT	18	12	−0.34	−3.65									
HIICT	18	17	−19.85	−40.18									
**Systolic Blood Pressure exercise (mmHg)**													
CG	18	12	−15.40	−7.75	3.48	0.038 ^2^	0.120	37.66	<0.001	0.394	6.24	0.016	0.098
MICT	18	12	−6.39	0.24									
HIICT	18	17	−3.06	−3.52									
**Diastolic Blood Pressure exercise (mmHg)**													
CG	18	12	−12.78	−5.10	17.4	<0.001 ^1,2^	0.405	8.37	<0.001	0.086	1.19	0.281	0.014
MICT	18	12	7.50	0.28									
HIICT	18	17	5.00	−0.68									
**Systolic Blood Pressure recovery (mmHg)**													
CG	18	12	−6.39	−4.70	0.0587	0.943	0.002	76.01	<0.001	0.589	13.332	<0.001	0.207
MICT	18	12	−5.84	−6.86									
HIICT	18	17	−6.95	−6.21									
**Diastolic Blood Pressure recovery (mmHg)**													
CG	18	12	1.39	−2.00	0.0540	0.947	0.002	73.137	<0.001	0.592	5.3698	0.025	0.097
MICT	18	12	1.11	−2.04									
HIICT	18	17	2.22	−0.30									
**Maximal speed reached during treadmill test (m/s)**													
CG	18	12	−0.08	−0.01	7.68	0.001 ^1,2^	0.231	10.28	0.023	0.134	0.317	0.575	0.005
MICT	18	12	0.51	−0.14									
HIICT	18	17	0.20	0.07									
**Body Mass Index (kg/m^2^)**													
CG	18	12	0.30	−0.05	6.99	0.002 ^2^	0.215	3.02	0.088	0.046	0.217	0.643	0.003
MICT	18	12	−0.10	1.47									
HIICT	18	17	−0.30	0.47									

ITT—Intention to treat, SD—Standard deviation, CG—Control Group, MICT—Moderate-Intensity Circuit Training, HIICT—High-Intensity Interval Circuit Training. ^1^ denotes significant differences in MICT compared to CG, ^2^ denotes significant differences in HIICT compared to CG, Statistically significant differences at *p* ≤ 0.05 are given in bold.

**Table 3:** Intra-group differences on HIICT, MICT, and CG.

Variables	Pre-training			Post-Training			*p*	95% CI for MD		Cohen’s d
	n	Mean	SD	n	Mean	SD		Lower	Upper	
**Maximal Oxygen Consumption estimated (mL/kg/min)**										
CG	18	26.8	5.17	12	26.00	4.86	0.288	−0.753	2.386	0.14
MICT	18	25.0	5.57	12	26.90	4.72	0.010	−3.378	−0.541	0.32
HIICT	18	26.1	5.63	17	29.50	5.75	<0.001	−3.968	−0.441	0.58
**Heart Rate peak (bpm)**										
CG	18	144.94	15.41	12	140.61	15.35	0.066	−0.316	8.983	0.26
MICT	18	150.78	22.64	12	150.06	17.94	0.770	−4.408	5.853	0.03
HIICT	18	148.83	17.76	17	144.11	20.37	0.125	−1.444	10.888	0.25
**Heart Rate recovery (bpm)**										
CG	18	79.54	8.13	12	80.35	8.13	0.669	−4.737	3.117	0.09
MICT	18	85.01	14.32	12	84.67	10.67	0.916	−6.204	6.871	0.02
HIICT	18	100.16	52.98	17	80.31	12.80	0.105	−4.629	44.327	0.36
**Systolic Blood Pressure exercise (mmHg)**										
CG	18	184.28	23.52	12	168.88	15.77	0.002	6.335	24.442	0.58
MICT	18	185.00	10.84	12	178.61	11.08	0.015	1.430	11.347	0.55
HIICT	18	178.61	9.67	17	175.55	6.15	0.213	−1.927	8.038	0.30
**Diastolic Blood Pressure exercise (mmHg)**										
CG	18	76.94	13.18	12	64.16	8.08	0.002	5.186	20.368	0.90
MICT	18	56.94	9.25	12	64.44	9.53	0.002	−11.699	−3.300	0.75
HIICT	18	58.33	6.86	17	63.33	6.18	0.003	−8.075	−1.925	0.69
**Systolic Blood Pressure recovery (mmHg)**										
CG	18	151.94	16.19	12	145.55	11.49	0.020	1.145	11.632	0.37
MICT	18	155.56	13.60	12	149.72	6.74	0.019	1.065	10.600	0.40
HIICT	18	151.11	11.70	17	144.16	5.49	0.028	0.845	13.043	0.57
**Diastolic Blood Pressure recovery (mmHg)**										
CG	18	73.33	7.27	12	74.72	5.27	0.462	−5.279	2.501	0.18
MICT	18	73.89	7.18	12	75.00	5.14	0.570	−2.258	2.703	0.14
HIICT	18	73.89	5.01	17	76.11	4.71	0.215	−5.862	1.418	0.42
**Maximal speed reached during treadmill test (m/s)**										
CG	18	4.94	0.80	12	4.86	0.79	0.448	−0.143	0.309	0.09
MICT	18	4.47	0.90	12	4.98	0.77	0.015	−0.544	−0.067	0.53
HIICT	18	5.21	0.82	17	5.41	0.89	0.001	−0.685	−0.203	0.23
**Body Mass Index (kg/m^2^)**										
CG	18	31.2	4.89	12	31.5	5.05	0.019	−0.52	−0.53	0.06
MICT	18	30.1	3.08	12	30.0	3.15	0.140	−0.02	0.29	0.03
HIICT	18	30.4	4.13	17	30.1	4.24	0.035	0.02	0.60	0.07

SD—Standard deviation, CG—Control Group, MICT—Moderate-Intensity Circuit Training, HIICT—High-Intensity Interval Circuit Training, Statistically significant differences at *p* ≤ 0.05 are given in bold.
